# Effect of ^18^F-DCFPyL PET on changes in management of patients with prostate cancer: a systematic review and meta-analysis

**DOI:** 10.3389/fmed.2024.1355236

**Published:** 2024-04-25

**Authors:** Hui Wang, HongMei Zhu, GuanNan Li, JiaoNa Dai, HeXiao Huang, Qiong Jia

**Affiliations:** ^1^Department of Nuclear Medicine, West China Hospital, Sichuan University, Chengdu, China; ^2^Department of Pediatrics, Peking University Third Hospital, Beijing, China

**Keywords:** ^18^F-DCFPyL, PET imaging, PET positivity, prostate cancer, management change

## Abstract

**Purpose:**

Prostate-specific membrane antigen (PSMA)-targeted imaging has gained increasing interest in its application in prostate cancer lesion detection. Compared with ^68^Galium (^68^Ga), ^18^Fluoride (^18^F)-labeled imaging agent has easier syntheses, lower price, and a longer half-time. 2-(3-{1-Carboxy-5-[(6-[^18^F]fluoro-pyridine-3-carbonyl)-amino]-pentyl}-ureido)-pentanedioic acid positron emission tomography (^18^F-DCFPyL PET) has been recently approved by the U.S. Food and Drug Administration. Several studies have proven its superiority to conventional imaging techniques in detecting prostate cancer lesions. However, the impact of ^18^F-DCFPyL PET on the management of patients with prostate cancer is not well established. Thus, we performed a systematic review and meta-analysis of available data to evaluate the impact of ^18^F-DCFPyL PET on the management of patients with prostate cancer.

**Methods:**

The PubMed, Embase, Scopus, and Cochrane databases were searched up to April 2024. Studies that reported the proportion of changes in management after ^18^F-DCFPyL PET was performed in patients with prostate cancer were included. The Grading of Recommendations Assessment, Development, and Evaluation system was used for the quality evaluation of the included studies. The proportion of changes in management was pooled using a random effects model. Meta-regression analyses were performed to assess the potential correlation between the PET positivity and management changes.

**Results:**

Fourteen studies (3,078 patients with prostate cancer) were included in our review and analysis. The pooled percentage of management changes was 43.5% (95% confidence interval [CI]: 33–54%). In patients with biochemical recurrent and for primary staging, the pooled percentage was 50% (95% CI: 39–60%) and 22% (95% CI: 15–29%), respectively. In the meta-regression analyses, PET positivity was detected as a significant predictor of management change (*p* = 0.0023).

**Conclusion:**

^18^F-DCFPyL PET significantly affects the management of patients with prostate cancer. Higher PET positivity rate significantly correlated with a higher proportion of management changes in patients with prostate cancer. However, more studies are still needed to confirm the important role of ^18^F-DCFPyL PET in the management of prostate cancer.

**Systematic review registration:**

https://www.crd.york.ac.uk/PROSPERO/#myprospero, CRD42022339178.

## Introduction

Prostate cancer is one of the most commonly diagnosed malignant diseases in men. It is projected that there will be approximately 2.3 million new cases and 0.7 million deaths by 2040 worldwide (1). Thus, it is important to accurately classify patients in the primary stage of the disease and closely monitor disease prognosis resulting from therapy decisions. Treatment decision-making process is usually significantly affect by many factors, such as extent and extraprostatic metastasis, clinicopathological status, and patient preferences (2). Besides, in patients with biochemical recurrence (BCR), it is appropriate to locate the lesions because its role as an indicator of disease progress is important for further disease management.

The most recent European Association of Urology (EAU) guidelines recommend at least one cross-sectional abdominopelvic imaging [computed tomography (CT) or magnetic resonance imaging (MRI)] and a bone scan for evaluating the extent of extra-prostatic disease in intermediate or high-risk prostate cancer (3). However, conventional imaging modalities detect malignancies mainly from morphological or osteoblastic activity data, and this becomes a major challenge when lesions develop in atypical locations, are small, or are from other benign pathologic processes such as fractures or infection. Once the BCR was detected following radical prostatectomy, only 11–14% of patients had a positive CT result for possible recurrent lesions (4). The pooled specificity of choline-based positron emission tomography (PET) imaging was proven higher than that of bone scan with fewer false-positive lesions (0.99 [95% CI: 0.93–1.00] vs. 0.82 [95% CI: 0.78–0.85]) (5). However, prostate-specific antigen (PSA) levels and kinetics dramatically affect the sensitivity of choline-based PET (6–8). Therefore, more accurate and advanced imaging modalities that can guide the management of prostate cancer patients are still needed in clinical practice.

Recently, prostate-specific membrane antigen (PSMA)-targeted PET tracers have gained increasing interest for imaging and selection of therapy in patients with prostate cancer. PSMA is a type II integral membrane glycoprotein produced by the prostatic epithelium, and is strongly overexpressed in prostate cancer cells (9, 10). Moreover, PSMA expression increases progressively in high-grade prostate tumor cells and metastatic lesions (11, 12). The most widely used PSMA ligand is ^68^Ga-PSMA-11 and rising clinical research is focused on its prostate cancer indications (13–17). However, its application has some disadvantages. ^68^Ga has a short half-life of 68 min and synthesis decreases as generators decay. In contrast, ^18^F has longer half-life, less positron energy, and possibly lower costs. Thus, ^18^F-labeled PSMA ligand is a promising alternative (18).

2-(3-{1-Carboxy-5-[(6-[^18^F]fluoro-pyridine-3-carbonyl)-amino]-pentyl}-ureido)-pentanedioic acid (^18^F-DCFPyL) is a widely used agent in clinical practice, and was recently approved by the U.S. Food and Drug Administration (FDA). It is a second-generation fluorinated PSMA-targeted PET radiotracer, and several studies have demonstrated that it holds promise in clinical application (19). Results from a study including 248 patients who were administered ^18^F-DCFPyL showed a rather high detection rate (17/29 scans, 59%) even with serum PSA value <0.5 ng/mL, and an overall detection rate of 86.3% (20). A recent meta-analysis by Pan et al. (21) showed that the pooled detection rates of ^18^F-DCFPyL-labeled PSMA PET/CT in prostate cancer was 92%. The pooled detection rate was 89% for PSA ≥ 0.5 ng/mL and 49% for PSA < 0.5 ng/mL.

Although the diagnostic performance of ^18^F-DCFPyL PET has been evaluated, its impact on changes in the treatment management of patients with prostate cancer has not been systematically reviewed. Therefore, in this systematic review and meta-analysis, we investigated the impact of ^18^F-DCFPyL PET on changes in the management of patients with prostate cancer.

## Materials and methods

This systematic review and meta-analysis was performed according to the Preferred Reporting Items for Systematic Reviews and Meta-Analyses (PRISMA) statement. The protocol was registered in the International Prospective Register of Systematic Reviews database (registration no. CRD 42022339178).

### Literature search

This work was performed according to the PRISMA statement. A systematic literature search was conducted through PubMed, Embase, Scopus, and Cochrane databases until April 2024. The search query was performed based on the following string of terms: (prostate OR prostatic) AND (^18^F-DCFPyL) AND (positron emission tomography OR PET) AND (impact OR change OR alter OR modification OR influence). All relevant studies were assessed, and bibliographies of the retrieved articles were evaluated for any relevant papers. Two reviewers independently performed the assessment and evaluation. Disagreements were reviewed and resolved by a third reviewer.

### Study selection

Two investigators (HW and HMZ) independently screened and assessed the titles, abstracts, and full texts of the eligible studies. Inclusion criteria were: (1) patients with primary staging (PS) or BCR prostate cancer (2) ^18^F-DCFPyL PET as “intervention,” (3) conventional imaging methods including CT, MRI, bone scan (BS) and other imaging modalities as “comparator,” (4) proportion of patients who have changed the disease management method as “outcome,” and (5) prospective or retrospective studies as “study design.” The exclusion criteria were as follows: (1) conference abstracts, review articles, editorials, and comments, and (2) overlapping cohort. Only the most recently published or largest study was included when there were multiple articles based on a similar population. Discrepancies were resolved by consulting a third investigator (QJ).

### Data extraction

Two investigators (HW and GNL) independently extracted data using a pre-specified method. Baseline characteristics, including authors, publication years, countries and institutions, study design, management plan, PET device, injected dose, uptake time, PET positive rate, and percentage of therapy alteration, were extracted from individual studies. Discrepancies were resolved by consulting a third investigator (QJ).

### Assessment of study quality

The quality of studies included in our study was evaluated by two independent reviewers according to the Grading of Recommendations Assessment, Development, and Evaluation (GRADE) system (22). They were assessed in four areas: study design, patient selection, publication, and indirectness. The studies included in our meta-analysis were not randomized trials (comparing management before and after ^18^F-DCFPyL PET) and were rated down because blinding was impossible. Quality assessment were performed by two independent reviewers (HXH and JND), and all discrepancies were generally resolved by consensus or decided by the senior reviewer (QJ).

### Data synthesis and analysis

In our meta-analysis, the impact of ^18^F-DCFPyL PET on the management of prostate cancer patients was the primary outcome, which represents the proportion of patients who underwent a change in therapy methods based on ^18^F-DCFPyL PET imaging findings. The secondary outcomes were the subgroup analyses for studies in patients with BCR and for PS.

After PET examination, 23.6% of cases underwent additional imaging, which cause overall 87.3% of management plan (23). In Metser et al. (24) and Liu et al.’s (25) studies, they have reported intend/recorded or actual/proposed treatment management, the recorded and actual numbers were analyzed.

Meta-analyses were conducted using R (version 4.2.1; R Foundation for Statistical Computing, Vienna, Austria, Package: meta). Heterogeneity was evaluated using Cochran’s Q test and Higgins and Thompson’s I^2^ test. Publication bias was assessed using funnel plots and Egger’s test, random effect was used for pooling, and meta-regression analyses were performed to investigate if PET positivity rate is the possible predictor of management change.

One study (26) reported the PET positivity rate from three readers, we used the mean of these values. One study (27) reported the PET positivity rate of local region, lymph node and metastasis, the number of metastatic detection rate was used. One study (28) included patients for PS and with BCR, the PET positivity rate was reported separately, and we used the mean value.

## Results

### Literature search

**Figure 1:**
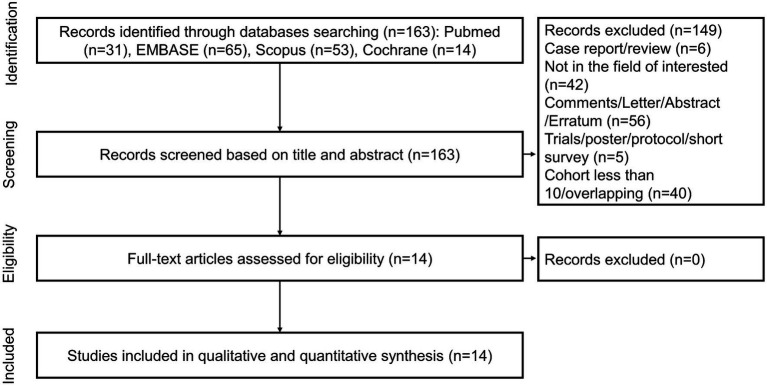
Flowchart of the article selection process.

A total of 163 unique records were identified from the literature search and screened for titles, abstracts, and full texts. With the removal of 149 papers after screening the titles and abstracts, 14 articles were included in full-text reviews, and all studies were ultimately selected. Figure 1 shows the study selection process in detail.

### Study characteristics

Study, patient, and PET characteristics are described in Tables 1–3 and Supplementary Table 1. The studies were conducted in Canada, the Netherlands, the United States, Spain, and Australia. There are 9 studies were prospective, and 5 were retrospective. The mean age range of the patients was 69–73 year. Serum mean PSA levels before ^18^F-DCFPyL PET were 0.32–15.8 ng/mL, respectively. The mean injected ^18^F-DCFPyL dose was 250–369 MBq, with mean uptake times ranging from 60 to 120 min. Reported patient management changes were BCR detection in 11 studies, PS in 4 studies, and both outcomes were reported in one study. PET positivity was reported in all studies, with values ranging from 15.5 to 87% (15.5% was reported as the detection rate of metastatic lesion, and for lymph node and local detection, the rate was 31, 93.1% respectively).

**Table 1:** Study characteristics.

Origin					Design			Management plan	
First author	Publication year	Patient enrollment	Institution	Country	Prospective	Multicenter	Data acquisition	Responding entity	Prior imaging
Chaussé	2020	July 2017–October 2018	Jewish General Hospital	Canada	P	No	Review	Multidisciplinary meeting	CT/MRI/BS
Liu	2020	January 2017–June 2018	London Health Sciences Centre and west University	Canada	P	Yes	Questionnaire	Referring physician	CT/mpMRI/BS
Meijer	2021	December 2016–December 2019	Prostate Cancer Network the Netherlands	Netherlands	R	Yes	Review	Referring physician	NR
Morris	2021	November 2018–August 2019	Memorial Sloan Kettering Cancer Center	USA	P	Yes	Questionnaire	Referring physician	CT, MRI, 11C-choline, ^18^F-fluciclovine PET, BS
Rousseau	2019	Interim analysis of an investigator-initiated clinical trial (NCT03181867)	BC Cancer	Canada	P	No	Questionnaire	Referring physician	CT/MRI/BS
Song	2020	May 2018–July 2019	Stanford University	USA	P	No	Review	Referring physician	CT/MRI/BS/^18^F-NaF/^18^F-Fluciclovine
Wondergem	2020	February 2018–April 2019	Noordwest Ziekenhuisgroep	Netherlands	R	No	Review	Multidisciplinary meeting	MRI/CT
Dias	2022	May 2018–December 2020	Mount Sinai Hospital & Women’s College Hospital,	Canada	P	No	Review	Multidisciplinary meeting	CT/BS/MRI
Metser	2022	December2018–September 2020	Mount Sinai Hospital & Women’s College Hospital,	Canada	P	Yes	Questionnaire	Referring physician	CT/BS
Zoghby	2023	August 2020–December 2021	University Hospital of Toledo	Spain	R	No	NR	NR	^18^F-choline PET/CT
Ng	2022	August 2018–July 2020	GenesisCare St. Vincent’s Hospital	Australia	P	Yes	Questionnaire	Referring physician	CT
Arafa	2023	August 2021–July 2022	University of Minnesota Masonic Cancer Center	USA	R	No	Review	NR	NR
Lucas	2023	March 2021–June 2022	University General Hospital	Spain	R	Yes	Review	Multidisciplinary meeting	CT/MRI/ ^18^F-Fluorocholine-PET/CT
Lager	2023	July–December 2020	Amsterdam University Medical Centers	Netherlands	P	Yes	Questionnaire	Multidisciplinary meeting	^18^F-fluoromethylcholine

BS, bone scintigraphy; CT, computed tomography; MRI, magnetic resonance imaging; NR, not reported; P, prospective; PET, positron emission tomography; R, retrospective.

**Table 2:** Patient characteristics.

First author	Patients (*n*)	Mean age (year)	Mean PSA (ng/ml)		Mean PSA-DT (mo)	Gleason score ≥ 8 (%)	Clinical setting	Primary treatment			On ADT (%)
			Initial	Pre-PET				RP (%)	RT (%)	Others (%)	
Chaussé	93	70.4	NR	4.57	NR	7*	BCR	30	27	43	47
Liu	79	73.2	9.2 ± 8.5	8.2 ± 10.6	16.2 ± 10.5	5	BCR	0	100	0	NR
Meijer	253	NR	NR	NR	NR	31.6	BCR	59	24.5	0	NR
Morris	208	68 (range 43–91)*	0.8 (range 0.17–98.45)*	1 (range 1–29)*	NR	26.4	BCR	49.5	14.9	0	27.9
Rousseau	130	69.1 ± 6.5	NR	5.20 ± 6.50	12.2 ± 11.8	36.5	BCR	72.3	34.6	0.8	47.4
Song	72	71.5 ± 7.2	NR	15.8 ± 83.2	NR	40	BCR	58	42	0	NR
Wondergem	160	71	NR	22.8(range 3.6–7,267)*	NR	55.6	PS	NR	NR	NR	NR
Dias	108	66 (IQR 61–73)*	NR	NR	NR	53	PS	0	0	0	0
Metser	1,289	71 (IQR 65–75)*	NR	1.2 (IQR 0.4–3.8)*	NR	NR	BCR	37.8	19	43.1	6.1
Zoghby	138	69.77 ± 7.54	NR	2.80 ± 4.83	7.34 ± 11.74	16.7	BCR	34.8	43.5	21.7	0
Ng	96	68.0 (95% CI 66.0–71.0)*	NR	0.32 (95% CI 0.28–0.36)*	NR	19.4	BCR	100	0	0	0
Arafa	189	PS: 69.0 (range 50.0–83.0)* BCR: 63.0 (range 45.0–80.0)*	PS: 18.8 (range 4.2–3740.0)* BCR: 7.5 (range 2.4–566.0)*	NR	BCR: 7.9 (range 0.3–118.0)*	PS:45.4 BCR:37.6	PS /BCR	80.7^#^	11.9^#^	5.5^#^	1.8^#^
Lucas	58	68.57 ± 7.95	24.73 ± 20.47	NR	31.78 ± 34.81	38	PS	0	0	0	0
Lager	205	70.0 ± 7.1	NR	0.93 ± 2.39^a^ 7.73 ± 14.32^b^	10.80 ± 12.74	18.7	BCR	73.2	26.8	0	0

ADT, androgen deprivation therapy; BCR, biochemical recurrence; NR, not reported; PS, primary staging; PSA, prostate-specific antigen; PSA-DT, prostate-specific antigen doubling time; RP, radical prostatectomy; RT, radiotherapy.

^*^Median.

^#^Systemic therapy.

^a^Patients treated with radical prostatectomy.

^b^Patients treated with radiation therapy.

**Table 3:** PET characteristics.

First author	Vendor	Model	Ligand	Mean dose (MBq)	Mean uptake time (min)	PET positivity (%)
Chaussé	General Electric Medical System	Discovery ST hybrid PET/CT	^18^F-DCFPyL	333 ± 37	60–90^#^	82
Liu	NR	NR	^18^F-DCFPyL	333 ± 33	NR	87.3
Meijer	NWZ: Siemens Healthineers UMC, NCI: Philips Healthcare	Biograph-16 TruePoint PET/CT Philips Ingenuity TF PET/CT Philips Gemini TF-II or Vereos Digital PET/CT	^18^F-DCFPyL	NWZ:290* (IQR 280–323) UMC:311* (IQR 301–322) NCI:197* (IQR 189–207)	120 (UMC, NWZ); 60 (NCI)	66
Morris	NR	NR	^18^F-DCFPyL	349 (277–410)*	79* (59–115)	61.5
Rousseau	GE Healthcare	Discovery PET/CT 600 or 690	^18^F-DCFPyL	369.2 ± 47.2	120.4 ± 1.5	84.6
Song	GE Healthcare	Discovery MI PET/CT scanner	^18^F-DCFPyL	338.8 ± 25.3	74.4 ± 10.4	85
Wondergem	Siemens Healthineers	Biograph-16 TruePoint PET/CT	^18^F-DCFPyL	328	120	Local: 98.1 N; M: 56
Dias	Siemens Healthcare	PET/CT or PET/MR	^18^F-DCFPyL	316 ± 15	115 ± 16	44 (N; M)
Metser	Siemens Medical Systems GE Healthcare Philips Medical Systems	Biograph mCT 16 Discovery VCT Gemini TF Big-Bore Discovery 710 Biograph mCT40	^18^F-DCFPyL	NR	NR	65.2
Zoghby	GE Healthcare	Discovery 5R/IQ hybrid PET/CT	^18^F-DCFPyL	4–5 MBq/Kg	100–120^#^	64.5
Ng	GE Healthcare	Discovery 710 PET/CT	^18^F-DCFPyL	250	120	46.9
Arafa	Siemens Healthcare	Biograph mCT-Flow 64HDTV PET/CT	^18^F-DCFPyL	333 ± 20%	60	PS(N; M): 46.2 BCR:62
Lucas	General Electric	Discovery 5R/IQ hybrid PET/CT	^18^F-DCFPyL	2–4 MBq/Kg	5–15; 120	Local: 93.1 N: 31 M: 15.5
Lager	NR	NR	^18^F-DCFPyL	316.56 ± 28.40	121.6 ± 7.9	58

CT, computed tomography; M, metastases; MRI, magnetic resonance imaging; N, pelvic lymph nodes; NCI, the Netherlands Cancer Institute; NR, not reported; NMZ, the Noordwest Ziekenhuisgroep; PET, positron emission tomography; UMC, Amsterdam UMC VU University.

^#^Range.

^*^Median.

### Quality assessment

**Figure 2:**
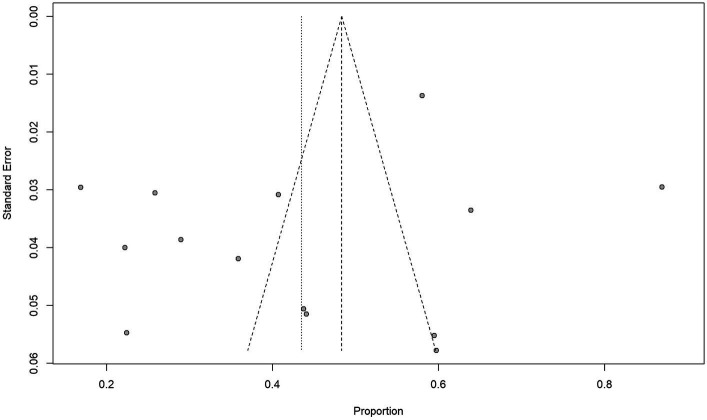
Funnel plot and Egger’s test suggest that the possibility of significant publication bias is low (*p* = 0. 2239).

In terms of bias, all included studies were rated down because blinding was impossible for therapeutic decisions based on conventional and ^18^F-DCFPyL PET imaging techniques. In terms of publication, two studies were rated down because of potential industry influence. Morris et al. (26) reported ^18^F-DCFPyL patent ownership and authors were employed in Progenics Pharmaceuticals. Liu et al. (25) reported personal fees from the industry and employment of a close family member by Roche Canada. All included studies have reported management changes and 5 studies were rated up due to a large effect size (> 50%) (23–26, 29). There was no other rating up or down in any of the studies selected for our review and analysis. Ultimately, the quality of evidence was high in 3 studies (23, 24, 29) and moderate in 11 studies (25–28, 30–36). Figure 2 shows the funnel plot and Egger’s test (*p* = 0.2239); there was no significant publication bias.

### Efficacy evaluation

**Figure 3:**
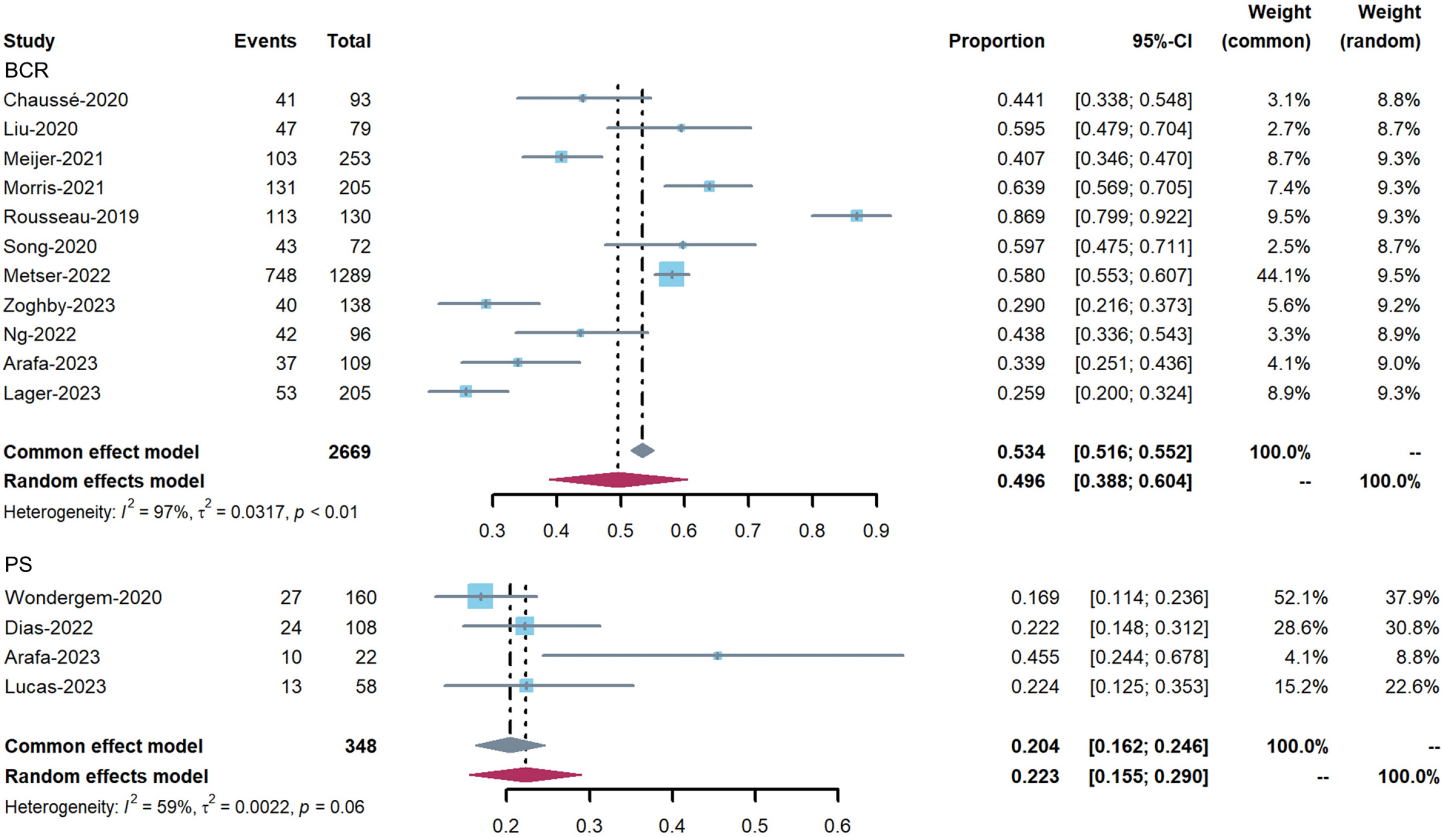
Forest plots showing the pooled proportion of management changes before and after ^18^F-DCFPyL PET, and classified into PS and BCR changes.

The impact of ^18^F-DCFPyL PET on patient management in all included studies, divided into BCR intent and PS intent, is shown in Figure 3. The proportion of management changes in individual studies varied from 17 to 87%. The pooled percentage of management changes was 50% (95% confidence interval [CI]: 39–60%) in patients with BCR (11 studies). Substantial heterogeneity was observed based on the Q test (*p* < 0.01) and Higgins and Thompson’s *I*^2^ statistic (*I*^2^ = 97%). For the four studies reporting patients for PS, the pooled proportion was 22% (95% CI: 15–29%), and heterogeneity was observed (Q test: *p* = 0.06; Higgins and Thompson’s *I*^2^ statistics: *I*^2^ = 59%). The overall pooled percentage of management changes 43.5% (95% CI: 33–54%, Q test: *p* < 0.01; Higgins and Thompson’s *I*^2^ statistics: *I*^2^ = 98%) (Supplementary Figure 1).

### Correlation between ^18^F-DCFPyL PET positivity and management change

**Figure 4:**
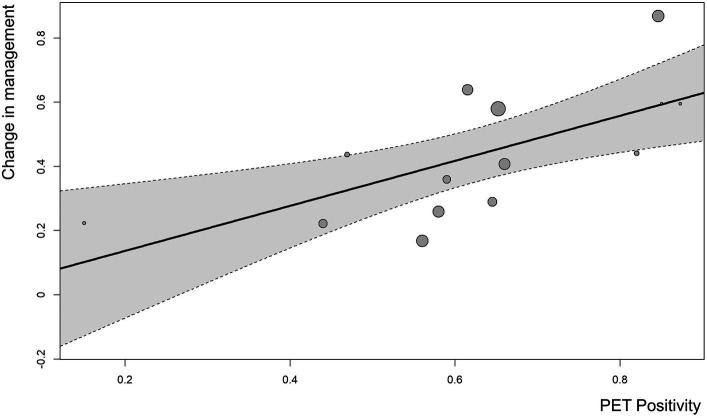
Bubble plot of the correlation between ^18^F-DCFPyL PET positivity and rate of management change using meta-regression analysis (*p* = 0.0023).

Figure 4 shows a clear tendency for greater ^18^F-DCFPyL PET positivity rate with a higher proportion of patient management changes (*p* = 0.0023). In addition, meta-regression analysis demonstrated that every 1% increase in PET positivity correlated with a 0.7% increase in management change.

## Discussion

In the current meta-analysis, we assessed the impact of ^18^F-DCFPyL PET imaging on the change in treatment methods of patients with prostate cancer. ^18^F-DCFPyL PET led to a pooled proportion of 43.5%, indicating that the performance of ^18^F-DCFPyL PET could affect the therapy decisions. In addition, we evaluated studies in which management change in patients with BCR detection or PS, and the pooled proportions were 50 and 22%, respectively.

A previous meta-analysis of the use of ^68^Ga-PSMA PET reported a similar pooled proportion (54%) of management changes, and the implemented and intended changes were 54 and 51%, respectively (37). These similar results were expected, which have confirmed the fact that PSMA-targeted molecular imaging has superior detection rates compared with conventional imaging modalities. Other novel PSMA ligands, such PSMA-1007 (38) and rhPSMA-7 (39), have been developed. However, due to limited research, the overall comparison among these tracers is lacking. Moreover, a positive correlation was observed between ^18^F-DCFPyL PET positivity and the proportion of management changes. Notably, Han et al. has reported that there was a 0.55% increase in management change for every 1% increase in ^68^Ga-PSMA PET positivity (37). In our study, a significant correlation between ^18^F-DCFPyL PET positivity and management change was confirmed, and the number of increase in management change for every 1% increase in ^18^F-DCFPyL PET was 0.7.

Several meta-analyses have reported that ^18^F-DCFPyL PET has relatively good sensitivity and specificity for the detection of both primary and metastatic prostate cancer lesions. The pooled detection rate of ^18^F-DCFPyL PET in prostate cancer patients was 92%. The pooled detection rate was 89% for PSA ≥ 0.5 ng/mL and 49% for PSA < 0.5 ng/mL (21). Another meta-analysis revealed that the pooled detection rate of ^18^F-DCFPyL PET in biochemically recurrent prostate cancer was 81% (95% CI: 76.9–85.1%). The pooled detection rate was 88.8% for PSA ≥ 0.5 ng/mL (95% CI: 86.2–91.3%) and 47.2% for PSA < 0.5 ng/mL (95% CI: 32.6–61.8%) (40). Moreover, in the included studies, ^18^F-DCFPyL PET was able to detect more lesions compared with conventional imaging, which is important to the patient because metastatic lesions could alter therapy methods. ^18^F-DCFPyL PET provides extra information in molecular level, which enables the detection of tiny metastatic lesions even in atypical location. There is increasing evidence that ^18^F-DCFPyL PET outperforms conventional imaging modalities in both PS (41) and BCR (20) of prostate cancer patients, and our results could potentially support the findings. Both ^68^Ga-PSMA-11 and ^18^F-DCFPyL were approved by the FDA, it is necessary to compare them comprehensively to provide evidence for selection of hospitals or institutions. Previous and the current studies have demonstrated a comparable detection rate of these two ligands. Thus, some institutions could consider the costs without harming patients’ management options. Moreover, new generations of ^18^F-labled PSMA-ligand have been proved with faster clearance and better tumor-to-background ratio, thus, ^18^F-labeled PSMA-ligand is a promising alternative option for prostate cancer patients.

This systematic review had several limitations. First, substantial heterogeneity was observed (*I*^2^ = 97, 98%). To our knowledge, baseline characteristics of patients (serum PSA level, Gleason score, risk stratification), clinical settings (PS, BCR), and types of primary treatment (radical prostatectomy, radiation therapy, systematic treatment) might be attributed to the results. It is already well known that different treatment patterns exist in the same region, likely because of differences in country, institution, specialty, and patient preference (37, 42–44). Besides, the conventional imaging modalities of the selected study are inconsistent, which could cause inequality among disease staging results. The lack of a reference standard for the interpretation of PSMA imaging could potentially cause bias, and more studies with histopathological confirmation are needed. Moreover, some reasons for the heterogeneity remain unexplained. Therefore, caution should be exercised when comparing and applying our pooled proportions. Although ^18^F-DCFPyL has been approved by the FDA, the number of related studies is limited. Thus, we could only include 14 studies, and none of them could be a blinded trial. Besides, 5 studies were retrospectively designed, and the risk of overestimating the pooled estimates and selection bias will increase. In addition, management changes between retrospective and prospective studies were not assessed because of the small sample size. Moreover, although it has proven that ^18^F-DCFPyL PET led to a change in management in approximately half of patients with prostate cancer, the correlation between alteration and outcomes or prognoses is still unknown. Finally, some studies lack post-operative histological validation and follow-up imaging; therefore, the interpretation of these results should be considered with caution. Thus, further clinical studies with standardized follow-up protocols and histological validation are needed to compare the outcomes of these tracers. The current study is based on ^18^F-DCFPyL PET; therefore, pooled evidence for PSMA-targeted imaging, including ^68^Ga-labeled PSMA is earnestly needed. Further studies are required to clarify this issue.

## Conclusion

The pooled proportion of prostate cancer patients experienced management changes was 43.5%, and ^18^F-DCFPyL PET has a significant impact on treatment options. Higher PET positivity rate is significantly associated with a higher proportion of management changes. Prospective studies with larger sample sizes and better follow-up are, therefore, urgently needed.

## Data availability statement

The original contributions presented in the study are included in the article/Supplementary material, further inquiries can be directed to the corresponding author.

## Author contributions

HW: Software, Writing – original draft, Conceptualization, Data curation, Methodology. HZ: Conceptualization, Data curation, Methodology, Writing – review & editing. GL: Conceptualization, Data curation, Methodology, Writing – review & editing. JD: Data curation, Methodology, Writing – review & editing. HH: Data curation, Investigation, Methodology, Writing – review & editing. QJ: Formal analysis, Funding acquisition, Investigation, Resources, Supervision, Writing – review & editing.
