# Supplementary material for: Effect of 18F-DCFPyL PET on changes in management of patients with prostate cancer: a systematic review and meta-analysis
Source: Front Med (Lausanne). 2024 Apr 25;11:1355236. doi: 10.3389/fmed.2024.1355236 (PMC11079165; doi:10.3389/fmed.2024.1355236)

Supplementary Table 1. Study characteristics

| First author | Publication year | Region of CT | Region of MRI | Number of patients with extra lesions in ^18^F-DCFPyL PET compared to CI | Location of additional lesions |
| --- | --- | --- | --- | --- | --- |
| Chaussé | 2020 | Chest, abdomen and pelvis | Pelvis | NR | Local, N, M |
| Liu | 2020 | Chest, abdomen and pelvis | Pelvis | 27 (34%) | local, oligometastatic, extensive metastases |
| Meijer | 2021 | NR | NR | NR | NR |
| Morris | 2021 | NR | NR | NR* | NR |
| Rousseau | 2019 | NR | NR | NR | NR |
| Song | 2019 | NR | NR | 26 (36%) | N, M |
| Wondergem | 2020 | NR | Prostate/pelvic region; spine | N: 39  M: 27 | N, M |
| Dias | 2022 | NR | Pelvis | Nodal: 25 (23%)  Distant: 13 (12%) | N, M |
| Metser | 2022 | abdominopelvic | NR | 615 (48%) | local, oligometastatic, extensive metastases |
| Zoghby | 2023 | NR | NR | 20 (14%) | Local, N, M^&^ |
| Ng | 2022 | Chest, abdomen, and pelvis | NR | Local: 19 (19%)  N: 18 (18%)  M: 6 (6%) | Local, N, M |
| Arafa | 2023 | NR | NR | NR | NR |
| Lucas | 2023 | NR | Pelvis | 4 (7%) | M^#^ |
| Lager | 2023 | NR | NR | 36 (18%) | Local, N, M^ab^ |

CI = conventional imaging; M= distant metastasis; MRI = magnetic resonance imaging; N = lymph node; NR = not reported; PET = positron emission tomography.

* Results from three readers was separated based on different modalities.

^#^ Compared ^18^F-DCFPyL PET and ^18^F-fluorocholine PET.

^&^ Compared ^18^F-DCFPyL PET and ^18^F-choline PET.

^a^ Compared ^18^F-DCFPyL PET and ^18^F-fluoromethylcholine PET.

^b^ Results from 3 readers.

Supplementary Figure 1. Forest plots showing the pooled proportion of management changes before and after ^18^F-DCFPyL PET.
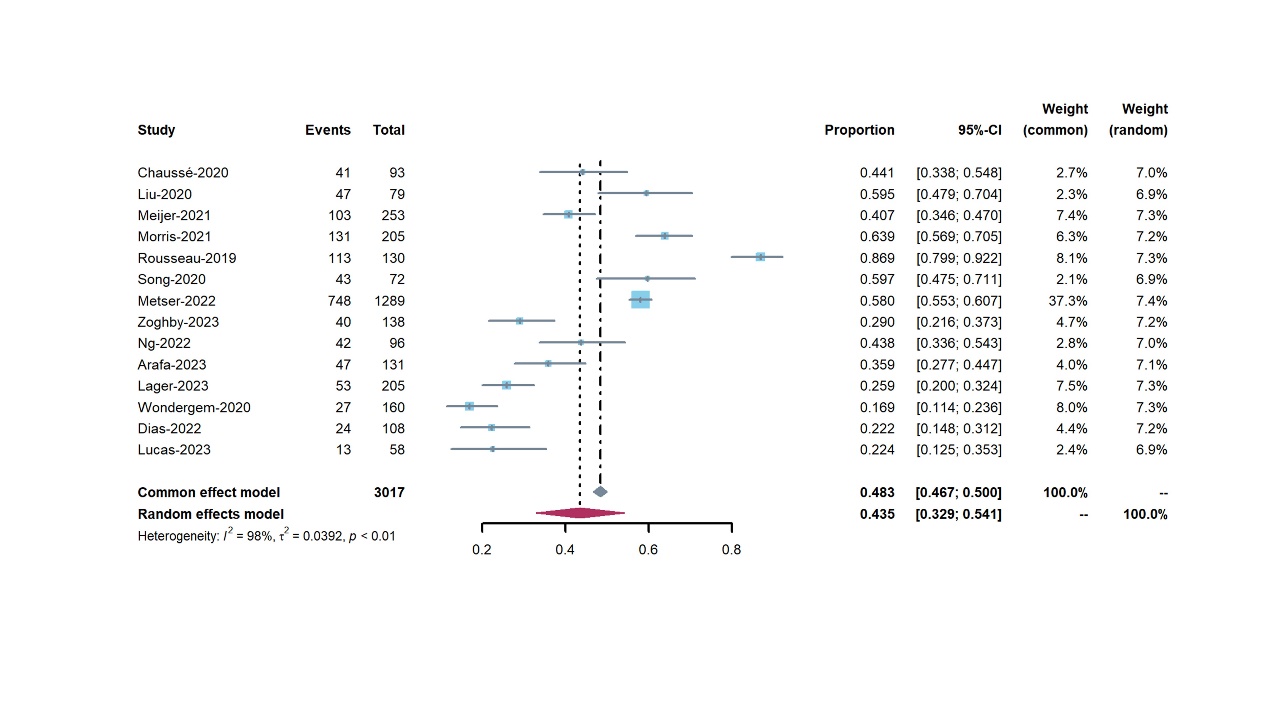

Supplement: Supplementary file 1 [file Data_Sheet_1.docx]
